# Effects of compression garment on muscular efficacy, proprioception, and recovery after exercise-induced muscle fatigue onset for people who exercise regularly

**DOI:** 10.1371/journal.pone.0264569

**Published:** 2022-02-28

**Authors:** Wei-Hsien Hong, Sui-Foon Lo, Hsin-Chieh Wu, Min-Chi Chiu

**Affiliations:** 1 Department of Sports Medicine, China Medical University, Taichung, Taiwan; 2 Department of Physical Medicine and Rehabilitation, China Medical University Hospital, Taichung, Taiwan; 3 Department of Industrial Engineering and Management, Chaoyang University of Technology, Taichung, Taiwan; 4 Department of Industrial Engineering & Management, National Chin-Yi University of Technology, Taichung, Taiwan; National Tsing Hua University, TAIWAN

## Abstract

Fatigue is a major cause of exercise-induced muscle damage (EIMD). Compression garments (CGs) can aid post-exercise recovery, therefore, this study explored the effects of CGs on muscular efficacy, proprioception, and recovery after exercise-induced muscle fatigue in people who exercise regularly. Twelve healthy participants who exercised regularly were enrolled in this study. Each participant completed an exercise-induced muscle fatigue test while wearing a randomly assigned lower-body CG or sports pants (SP); after at least 7 days, the participant repeated the test while wearing the other garment. The dependent variables were muscle efficacy, proprioception (displacements of center of pressure/COP, and absolute error), and fatigue recovery (muscle oxygen saturation/SmO_2_, deoxygenation and reoxygenation rate, and subjective muscle soreness). A two-way repeated measure analysis of variance was conducted to determine the effect of garment type. The results indicated that relative to SP use, CG use can promote muscle efficacy, proprioception in ML displacement of COP, and fatigue recovery. Higher deoxygenation and reoxygenation rates were observed with CG use than with SP use. For CG use, SmO_2_ quickly returned to baseline value after 10 min of rest and was maintained at a high level until after 1 h of rest, whereas for SP use, SmO_2_ increased with time after fatigue onset. ML displacement of COP quickly returned to baseline value after 10 min of rest and subsequently decreased until after 1 hour of rest. Relative to SP use, CG use was associated with a significantly lower ML displacement after 20 min of rest. In conclusion, proprioception and SmO_2_ recovery was achieved after 10 min of rest; however, at least 24 h may be required for recovery pertaining to muscle efficacy and soreness regardless of CG or SP use.

## Introduction

The benefits of regular physical activity are irrefutable, with substantial evidence indicating enhanced cardiovascular, musculoskeletal, metabolic, and mental health [1]. According to a Sports Administration survey, in Taiwan, the proportion of the population who regularly exercise (at least 3 times per week) increased from 24.4% in 2009 to 33.5% in 2018 [2]. With the prevalence of exercise, exercise-induced muscle damage (EIMD) should be studied [3]. A study reported that up to 70% of people with exercise habits and race runners sustained injuries from musculoskeletal overtraining in a year; lower limb injuries were the most common with rates ranging from 19.4% to 79.3% [3], and fatigue was the main cause of EIMD. Although performance outcomes are usually the focus of athletes and sports scientists [4], they are also concerned about identifying effective strategies for athletes to quickly recover from fatigue after exercise and for reducing the EIMD-related negative symptoms.

Several recovery strategies can help reduce the effects of EIMD or accelerate recovery from fatigue; they include cold therapy [5], use of antioxidants supplements [6], and use of compression garments (CGs) [7]. Studies have reported that relative to other recovery strategies, CG use may provide more advantages in improving post-exercise recovery [7, 8]. However, to date, the body of evidence is inconclusive; the two studies that compared CG use with other recovery methods reported different conclusions [9, 10]. CG use was effective at enhancing the rate of recovery of creatine kinase following EIMD onset [9]. However, some study suggested that cold water immersion was more effective than CG use in enhancing recovery [10]. Studies have examined CG use during and up to 24 or 48 h after an exercise program; they reported that CG use provided the greatest benefit in improving recovery from muscle soreness but did not produce consistent findings regarding improvements to creatine kinase recovery [7, 11, 12]. The aforementioned studies have not verified whether fatigue was actually achieved through exercise-induced muscle fatigue programs. Furthermore, most CG-use studies performed posttests at 24, 48, or 72 h after initial testing [11, 12], whereas few studies have focused on recovery within 1 h after exercise-induced muscle fatigue onset.

Peripheral fatigue was reported to have multiple etiologies, and a potential cause is oxygen availability [13]. The measurement of muscle oxygen saturation (SmO_2_) with noninvasive near infrared spectroscopy technology was used as a possible indicator of fatigue to provide real-time physiological feedback [13], and it was validated for use in evaluating dynamic exercises performed by adults [14]. CG use may contribute to recovery by increasing venous return and tissue oxygenation [15]. The reoxygenation rate of SmO_2_ (unit, %/s) is evaluated according to the reoxygenation slope. A higher reoxygenation rate indicates greater O_2_ delivery relative to O_2_ consumption, which increases blood flow during recovery [16]. Few studies have explored the effects of CG use on deoxygenation rate during the fatigue period and reoxygenation rate during the recovery period.

The somatosensory system contributes substantially to balance control, and proprioception is a key component of the somatosensory system, which uses information input from mechanoreceptors in the soft tissues of joints and muscles to transmit information to the central nervous system regarding the motion and position of the body in space [17]. The overall compression achieved through CG use acts as a mechanically supportive framework that is sensitive to body movements and can activate individual cutaneous mechanoreceptors that would not have been activated otherwise. Therefore, CG use can enhance the perception of somatosensory information and influence balance control by reducing body sway during quiet standing [18]. The single-leg stance with eyes closed (thereby removing visual information) is commonly used to assess an individual’s proprioceptive abilities, and it has a good interrater reliability (r = 0.87) [19]. Joint position sense (JPS) is another commonly used measure of proprioception, and it involves detecting the spatial position of one’s body [20]. Studies have speculated that CG use enhances JPS by reducing muscle fatigue [21, 22]. However, conflicting results were reported regarding how and whether tissue compression induced through CG use affects knee JPS [23]. Therefore, the purpose of the present study was to clarify the effects of CG use on muscular efficacy, proprioception, and recovery after exercise induced muscle fatigue in people who exercise regularly.

## Materials and methods

### 1. Subjects

Twelve healthy participants with regularly exercise habit (6 men and 6 women) were enrolled in the present study; their average age, height, and weight were 22.8 ± 2.2 years, 169.6 ± 7.2 cm, and 67.2 ± 12.7 kg, respectively. Regular exercise habit was defined as engagement in exercise or fitness activities at least 3 times per week and for 30 min per exercise session. Individuals were included that they did not have a lower limb injury within previous 6 months. Prior to the study, none of the participants had worn a CG on a regular or consistent basis. The participants were requested to avoid heavy exercise before the day come to testing. Individuals were excluded if they had a history of musculoskeletal injury and inflammatory disorders. The experimental procedure was approved by the Ethics Committee of China Medical University Hospital. The eligible participants were informed of the experimental procedures and precautions, and they were asked to sign an informed consent form.

The sample size calculations (G*Power 3.1.7) were based on those of another study [22]. The power analysis that was performed using repeated measures ANOVA indicated a total sample size of 12, with an assumed type I error of 0.05 and power of 0.80. Although the power analysis revealed that the number of participants was sufficient for detecting differences between the results achieved with the garment types over time, increasing the sample size can increase the statistical power of the experiments.

### 2. Experimental instruments

#### (1) Force plate

An AMTI force plate (Model OR 6-5-1000, Advanced Mechanical Technology, Watertown, MA, USA), which was sampled at 1000 Hz was used to collect jump height and center of pressure (COP) data to calculate power and proprioception control.

#### (2) Muscle oxygen monitor

A MOXY monitor (Moxy, Fortiori Design, MN, USA) is a portable and wireless muscle oxygen monitor that can continuously measure SmO_2_%. Noninvasive near infrared spectroscopy uses near-infrared light between 670 and 810 nm to penetrate the muscle fiber and determine the ratio of oxyhemoglobin concentration to total hemoglobin concentration in the muscle; this is achieved by determining the difference in the absorbencies between oxygenated/deoxygenated hemoglobin and myoglobin. SmO_2_% is outputted as a percentage using the following equation:

$$SmO_{2}\%=\left(\frac{oxygenated\;hemoglobin+myoglobin}{total\;amount\;of\;hemoglobin+myoglobin}\right)\times 100$$

MOXY sensors were placed on the quadriceps and the gastrocnemius medialis (GM) of the dominant leg. Electrodes were placed per surface electromyography for the non-invasive assessment of muscles guidelines [24]. Because a CG causes compression and tightening, after an electrode pad was attached to a muscle surface, the pad was secured with medical mesh and breathable tape to prevent slipping after sweating.

### 3. Lower body CG

**Fig 1:**
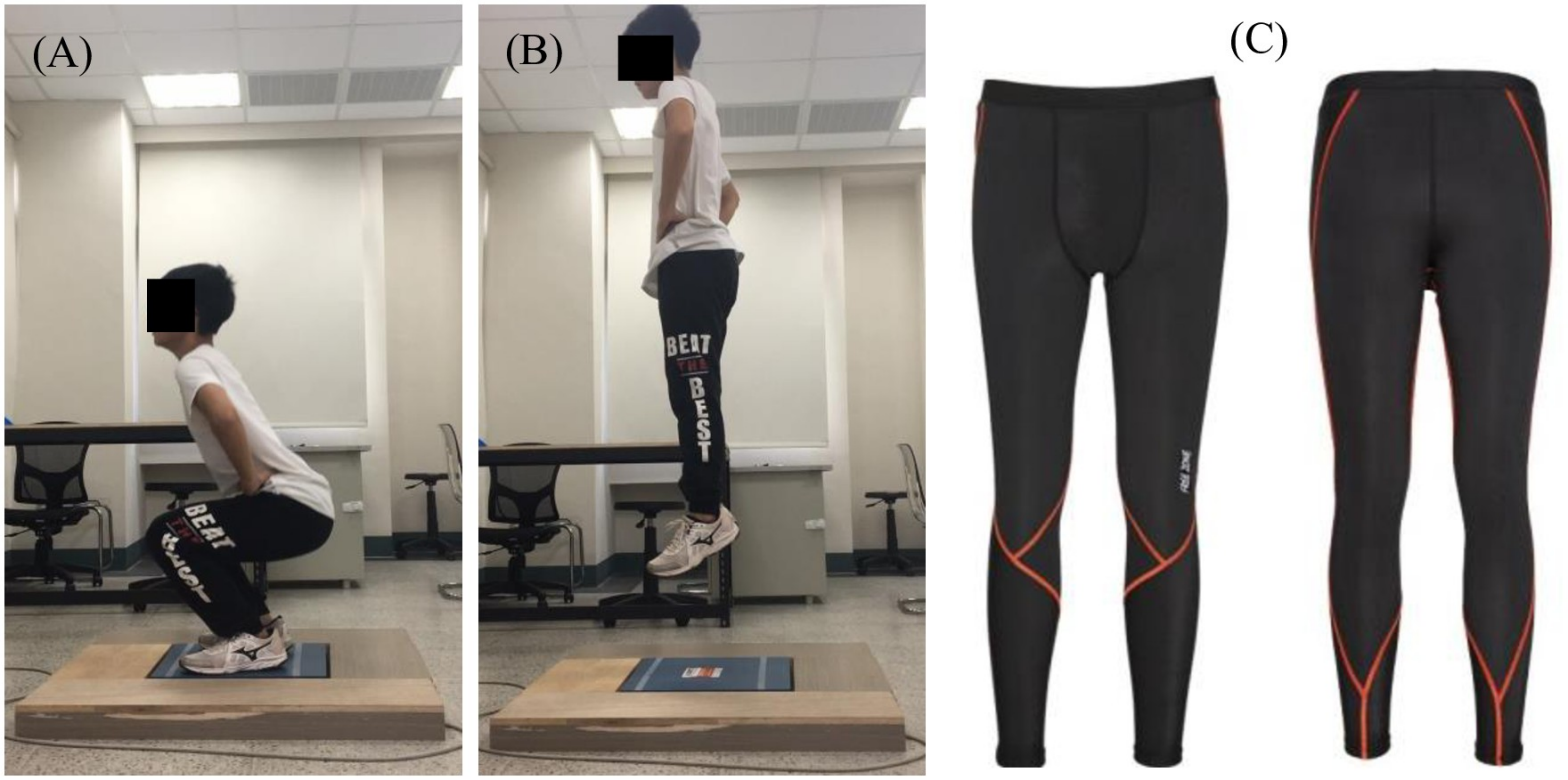
During the execution of countermovement jumps, participants (A) flexed their knees such that their thighs are approximately parallel to the ground, (B) and a maximal vertical jump was then performed. (C) Lower body compression garment (FreeZone). Image source: www.free-zone.com.tw.

The lower body CG (FreeZone CG, BOURTEX, Taiwan) is made from 70% polyester fiber and 30% elastic fiber; its pressure distribution is 27.3 mmHg from the ankle, 24.4 mmHg from the ankle to the knee, 21.5 mmHg from the knee, and 16.8 mmHg from the knee to the thigh (Fig 1). The CGs were allocated to each participant according to the manufacturer’s size guidelines. General sport pants do not have a pressure effect, and they generally do not interfere with measurement taking. The participants were required to wear the same shoes for both the CG- and SP-use tests.

### 4. Fatigue protocol

Muscular fatigue in the lower limbs is measured during the performance of a countermovement jump (CMJ) on a force plate [25]. A participant first initiates a jump while standing on the platform with legs extended and hands on the waist to improve control and reduce the effects of arm swings; next, the participant follows the metronome and jumps at a rate of 45 times per min. The jumping rate choice is referred to previous study [26], and participants subjectively assented that the jumping rate of 45 bpm were acceptable and reasonable to induce individual fatigue feelings. For the jumping and landing components, a participant’s legs were required to be flexed such that the thighs are approximately parallel to the ground. Each participant practiced his or her jumping technique and verified it prior to testing to ensure best effort during the fatigue test (Fig 1). Participants were regarded as fatigued when the Borg rating of perceived exertion (RPE) reached level 17 or higher, and they could no longer maintain their jump height or keep in pace with the metronome. Throughout the fatigue test, a muscle oxygen monitoring system was used to monitor muscle oxygen concentration.

### 5. Measurement

#### (1) Muscular efficacy

Power, which relates to muscular efficacy, is defined as the ability of skeletal muscle to rapidly generate force [27]. The CMJ data that were collected through the force plate were processed using AMTI software (Version 3.05.4) to calculate takeoff and landing contact time. Body weight (BW) and the standard deviation of BW were calculated using the average of the first 500 ms of unfiltered data on the Z-axis. Triaxial (X, Y, Z) data were processed through a 10-Hz 4th-order low-pass Butterworth filter. A vertical force of <20 N indicated the takeoff point, and a vertical force of >20 N indicated the landing contact-time point; these points were then used to calculate flight time (t). Jump height was calculated using the formula 1/8gt^2^ (t, flight time; g, gravity constant) [28]. The aforementioned values were substituted into the following formula to calculate the peak power:

$$\text{Peak}\;\text{power}=\left(51.9\right)\times \text{jump}\;\text{height}\;\left(\text{cm}\right)+48.9\times \text{body}\;\text{mass}\;\left(\text{kg}\right)-2007\;({\text{r}}^{2}=0.78)$$

[29]. The obtained value represents the power of the legs.

#### (2) COP displacements and JPS

The force platform data were smoothed using a 10-Hz 4th-order zero-lag low-pass Butterworth filter, and the COP data were high-pass filtered at a cut-off frequency of 0.1 Hz.

The COP displacements in the anteroposterior (AP) and mediolateral (ML) were calculated per the standard formulas (which are described in the force plate manual) as follows:

$${COP}_{AP}=\frac{M_{AP}}{F_{Z}}$$

$${COP}_{ML}=\frac{M_{ML}}{F_{Z}}$$

where M_AP_ and M_ML_ indicate the moments in the AP and ML directions, respectively, and F_Z_ indicates force in the vertical direction.

JPS was defined through absolute error, that is, the difference between the perceived angle and target angle; no directional bias is involved. Each participant was asked to actively flex their knee to 45° (with their eyes open) from a standing position and to hold this position for 5 s; the participant was then allowed to memorize the position and knee flexion angle (defined as the target angle). The participant then returned to a standing position, and after a 7-s interval, he or she then attempted to reproduce the angle with his or her eyes closed. The participant moved his or her lower limb through active contraction at an angular velocity approximating 2°/s and stopped and held a position for 5 s when he or she perceived that the target angle was achieved. The holding time applied in the present study was identical to those used in previous studies [30].

#### (3) Reoxygenation and deoxygenation rate

**Fig 2:**
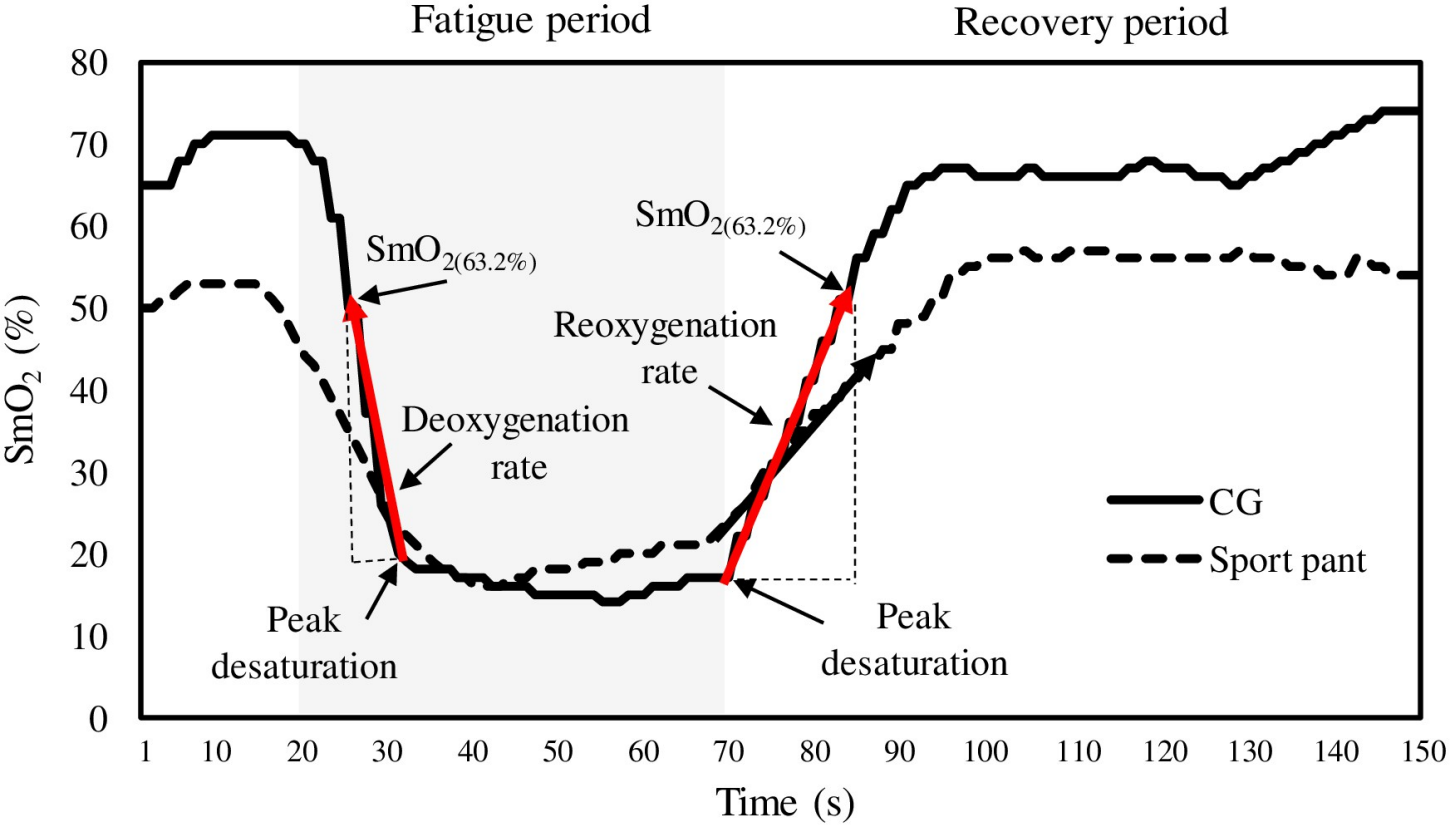
Definition of deoxygenation rate during fatigue period and reoxygenation rate during recovery period; CG, compression garment.

The definitions and equations are adjusted with reference to another study [31]. Fig 2 presents the SmO_2_ curve from the fatigue to initial recovery period. According to Fig 2, the reoxygenation slope of SmO_2_ = (SmO_2(63.2%)_ − SmO_2(PD)_)/(T_63.2%_ − T_PD_). SmO_2(PD)_ was the SmO_2_% value at peak desaturation during the recovery period with a related time of T_63.2%_; SmO_2(63.2%)_ was the SmO_2_% value at 63.2% of the baseline value during the recovery period with a relative time of T_PD_. The slope represents the index of reoxygenation rate (unit: % /s), and a higher reoxygenation rate indicates a greater O_2_ supply relative to O_2_ demand.

The deoxygenation slope of SmO_2_ = (SmO_2(PD)_ − SmO_2(63.2%)_)/(T_PD_ − T_63.2%_), and SmO_2(PD)_ was the SmO_2_% value at peak desaturation during the fatigue period with a related time of T_63.2%_; SmO_2(63.2%)_ was the SmO_2_% value at 63.2% of the baseline value during the fatigue period with a relative time of T_PD_.

#### (4) Muscle soreness scale

A visual analog scale (VAS) was used to assess the level of pain/soreness felt in the tested quadriceps and GM muscles. The participants were asked to place a mark on a horizontal 100-mm line to indicate the maximum amount of pain/soreness experienced during fatigue onset and at 10 min, 20 min, 30 min, and 1 h after completing the fatigue protocol. The starting point (0 mm) and endpoint (100 mm) on the 100-mm line corresponded to the “no pain/soreness” and “worst pain/soreness imaginable” pain levels, respectively.

### 6. Experimental procedure

The present study adopted a randomized crossover design. The same group participants were completed two trials with a randomly process, either wearing a lower-body CG or a pair of general sport pants (SP) (i.e., control) could be assigned in the first trail. Secondary trial would be executed with the other condition after one week later. Each participant completed an exercise-induced muscle fatigue protocol involving CMJs while wearing a CG and SP. Measurements were taken at baseline (before CMJs), fatigue onset, and after 10 min, 20 min, 30 min, and 1 h of rest (following fatigue onset).

First, all participants provided written informed consent, after which the experimenter collected their demographic data. Before each test, the participants performed a warm-up routine that comprised the stretching of lower limbs muscles and 2 min of jumping exercises. Next, MOXY sensors were placed on the GM and quadriceps of a participant’s dominant leg.

At the start of a test, a participant performed three vertical CMJs on a force plate and rested for 20 s between each repetition. All jumps were performed with hands on the waist. The participants were asked by the same experimenter to jump as high as possible for each jump. During a CMJ, the participants flexed their knees such that their thighs were approximately parallel to the ground; this position was held for 2 s before a maximal vertical jump was performed [32]. A CMJ was performed with a rapid descent to a self-selected depth, which was immediately followed by a maximal ascent until a straight standing posture was achieved [33].

Next, each participant performed a 10-s test by using their dominant limb to assume a single-leg stance with eyes closed on the force plate. The tested limb was in full knee extension, the participants had their hands on their waist, and the foot of the non-tested limb was positioned above the test surface without touching the tested limb [34]. The participants performed this test thrice with the dominant limb and rested for 1 min between each test. A test was stopped and the stance time for a trial was recorded when the foot of the non-tested limb touched the testing surface or tested limb or when a hand left the waist. A test was regarded as acceptable if a participant maintained the required position for a minimum of 5 s.

Thereafter, the participants performed the JPS test. Three tests were performed, and the difference between the perceived and target angles were noted for each test. A universal 360° manual goniometer (RBMS, USA) was used to measure knee joint angle. The good reliability and validity of the aforementioned reproduction procedure and manual goniometer were verified by other researchers [35]. After the JPS test was completed, each participant was required to assess the muscle soreness levels of both muscles by using the VAS.

After the baseline tests were completed, the fatigue protocol was executed; the participants rested for 3 min after the fatigue test and returned to the force plates to perform another three maximal vertical jumps. Measurements were also taken after 10 min, 20 min, 30 min, and 1 h of rest; the tested and assessed items for each time point were identical to those examined at baseline, and SmO_2_ data were collected throughout the entire experimental process by using the MOXY monitor.

### 7. Statistical analysis

The dependent variables were muscle efficacy (jump height and power), proprioception (AP and ML displacements and absolute error), and fatigue recovery (SmO_2_, reoxygenation and deoxygenation rate, and muscle soreness using the VAS. Statistical analyses were performed using SPSS 21.0 for Windows (SPSS, Chicago, IL, USA). A two-way repeated measure analysis of variance (ANOVA) was conducted to determine the effect of garment type (CG use vs. SP use) over six time points (before CMJs, fatigue onset, and after 10 min, 20 min, 30 min, and 1 h of rest), and their interactions with respect to the variables of muscular efficacy, proprioception, and fatigue recovery. When the main effects were detected to have significant differences, Bonferroni corrected paired *t* tests were performed as post-hoc tests. In addition, a paired *t* test was performed to compare the differences between the garment types for each time point and for deoxygenation and reoxygenation. The statistical significance was recognized when *p* < 0.05.

## Results

**Fig 3:**
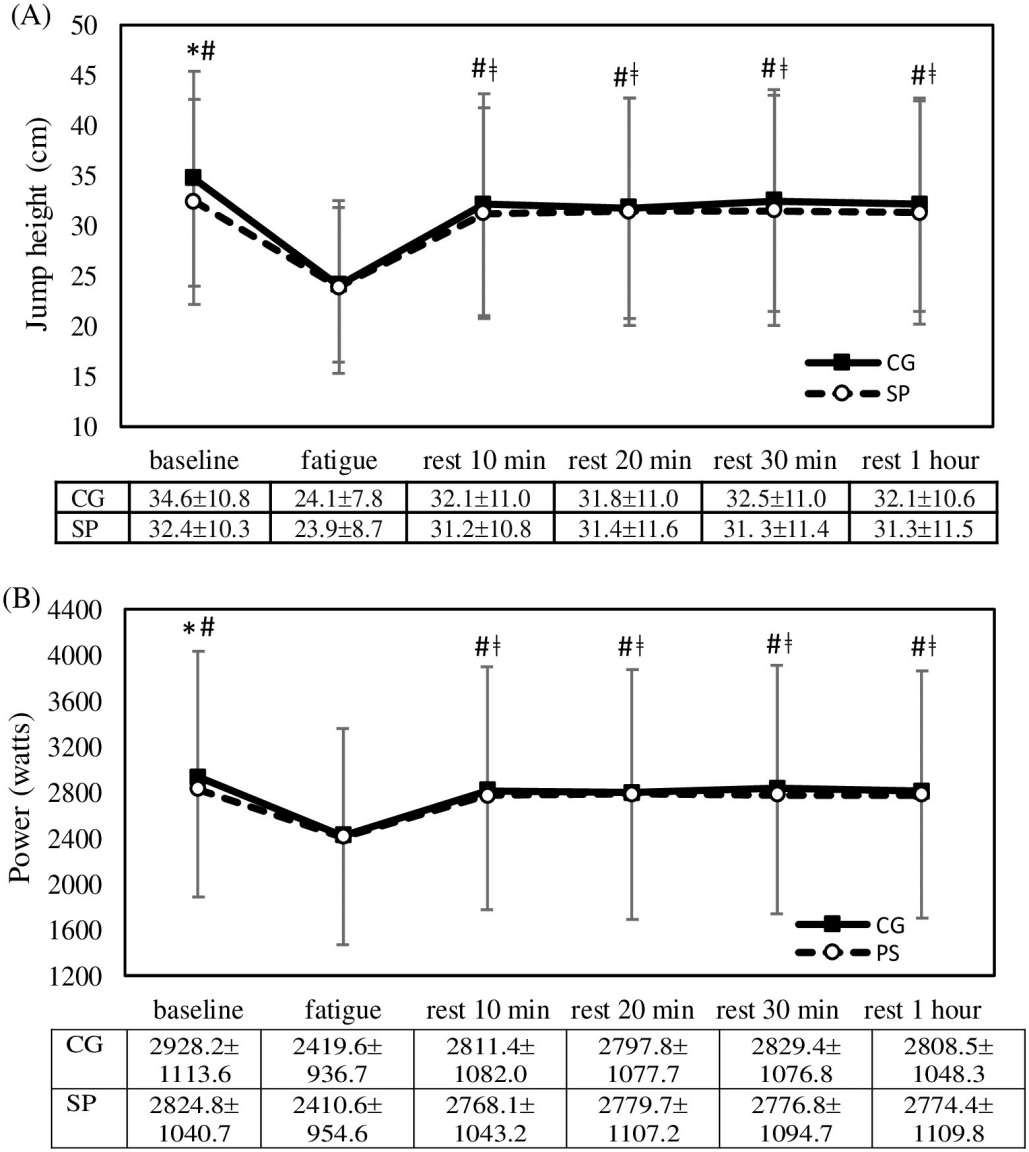
Comparisons of (A) jump height and (B) power between garment type and time course of change. Error bars are standard deviation; CG, compression garment; SP, sport pants; *, p<0.05, CG compared with SP; #, p<0.05, compared with fatigue onset; ǂ, p<0.05, compared with baseline.

Fig 3 presents the time course of change in jump height and power during CMJs, and it indicates similar results. Significant main effects were observed for garment type (jump height: F = 26.7, *p* < 0.001; power: F = 28.6, *p* < 0.001) and time point (jump height: F = 37.9, *p* < 0.001; power: F = 38.8, *p* < 0.001), but no significant interaction (jump height: F = 1.21, *p* = 0.316; power: F = 1.5, *p* = 0.209) was observed. Jump height and power significantly decreased at fatigue onset (*p* < 0.05) and remained significant lower than their baseline values at all-time points during the rest period (*p* < 0.05); however, no significant difference between jump height and power was observed. At baseline, jump height and power were significant greater with CG use than with SP use (jump height: t = 2.62, *p* = 0.024; power: t = 2.85, *p* = 0.014).

Table 1 presents the time course of change in AP and ML displacements for the single-leg stance with eyes closed and the absolute error of JSP. For AP displacement, the ANOVA did not reveal significant main effects for garment type (F = 1.69, *p* = 0.220) and time point (F = 1.21, *p* = 0.316) and significant interactions (F = 1.02, *p* = 0.414). For ML displacement, significant main effects were observed for garment type (F = 11.0, *p* = 0.010) and time point (F = 25.8, *p* < 0.001), but no significant interaction (F = 2.27, *p* = 0.058) was observed. For ML displacement, a significant increase was observed at fatigue onset (*p* < 0.05), but no significant difference was observed between baseline and the time points during the rest period. For ML displacement, no significant difference between garment types was observed at baseline; however, relative to SP use, CG use led to a significantly smaller ML displacement after 20 min of rest (20-min rest: t = 2.47, *p* = 0.031; 30-min rest: t = 3.71, *p* = 0.003; 1-h rest: t = 2.95, *p* = 0.013).

**Table 1:** Comparisons of proprioception variables between garment type and time course of change (mean±SD).

Time points Parameters	Baseline	Fatigue	Rest 10 min	Rest 20 min	Rest 30 min	Rest 1 hour
AP displacement (cm)						
CG	3.80±1.28	4.04±1.39	3.80±1.12	3.75±0.80	3.61±0.75	3.33±0.76
SP	4.18±1.50	3.91±0.84	4.41±1.87	3.46±1.02	4.28±1.97	4.04±1.05
CG vs SP *p* value	*p* = 0.386	*p* = 0.735	*p* = 0.309	*p* = 0.459	*p* = 0.321	*p* = 0.030*
ML displacement (cm)						
CG	5.92±1.07^#^	7.80±0.82	6.27±1.28^#^	5.52±0.95^#^	5.35±1.24^#^	5.12±1.20^#^
SP	6.18±1.10	8.02±1.05	6.55±1.17	6.27±1.02	6.25±1.08	5.75±1.13
CG vs SP *p* value	*p* = 0.374	*p* = 0.246	*p* = 0.073	*p* = 0.031*	*p* = 0.003*	*p* = 0.013*
Absolute error (°)						
CG	2.0±1.2^#^	6.0±3.5	3.1±2.4^#^	1.9±1.5^#^	1.6±1.0^#^^,^^a^	1.0±0.7^#^^,^^a^
SP	1.9±2.7	5.9±3.8	2.1±1.5	1.7±1.5	1.4±1.2	1.4±1.5
CG vs SP *p* value	*p* = 0.916	*p* = 0.819	*p* = 0.296	*p* = 0.718	*p* = 0.770	*p* = 0.426

^#^: *p* < 0.05 compared with fatigue onset; AP, anteroposterior; ML, mediolateral; CG, compression garment; SP, sport pants;

*, p<0.05, CG compared with SP;

^a^, p<0.05, compared with rest 10 min; SD, standard deviation.

**Fig 4:**
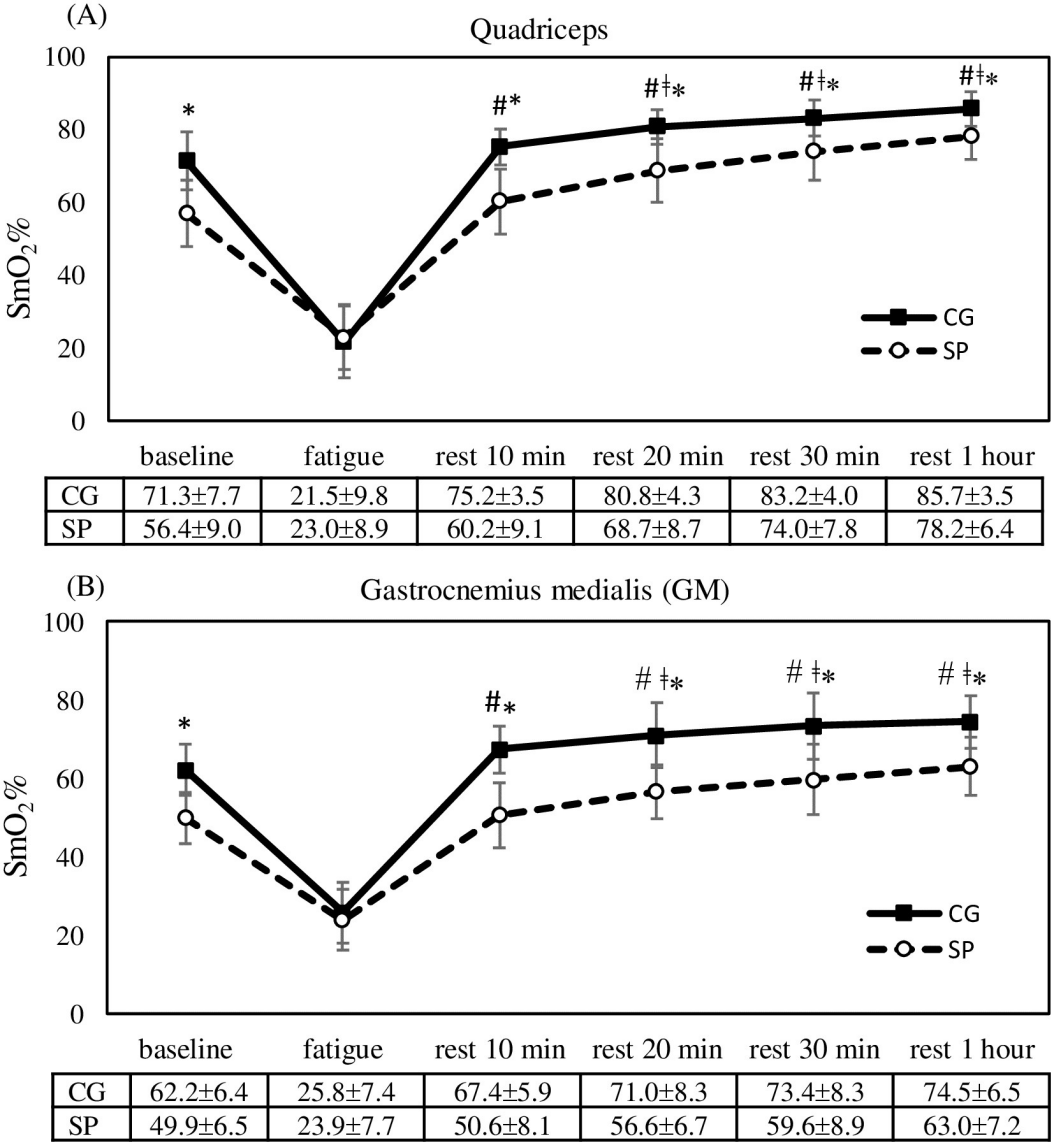
Comparisons of SmO_2_% in (A) quadriceps and (B) GM between garment type and time course of change. Error bars are standard deviation; CG, compression garment; SP, sport pants; *, p<0.05, CG compared with SP; #, p<0.05, compared with fatigue onset; ǂ, p<0.05, compared with baseline.

For absolute error, the ANOVA revealed a significant main effect for time point (F = 20.7, *p* < 0.001), but no significant effect was observed for garment type (F = 0.002, *p* = 0.976) and interaction (F = 0.443, *p* = 0.816). Absolute error significantly increased at fatigue onset (*p* < 0.05), and a greater absolute error was observed after 10 min of rest than after at 30 min and 1 h of rest (*p* < 0.05); however, no significant difference between the time points was observed after 20 min of rest (Table 1). Fig 4 presents time course of change in SmO_2_%. Significant main effects were observed for garment type (quadriceps: F = 32.5, *p* < 0.001; GM: F = 38.2, *p* < 0.001) and time point (quadriceps: F = 64.9, *p* < 0.001; GM: F = 59.4, *p* < 0.001), and a significant interaction (quadriceps: F = 10.5, *p* < 0.001; GM: F = 7.2, *p* < 0.001) was also observed. A significant decrease in SmO_2_% was observed at fatigue onset (*p* < 0.05); no significant differences were observed between baseline and after 10 min of rest; however, when the rest time became longer, a higher SmO_2_ was observed (*p* < 0.05). At baseline, SmO_2_% was significantly higher with CG use than with SP use (t = 2.85, p = 0.014), and similar results were observed at each time point after 10 min of rest (*p* < 0.05).

**Fig 5:**
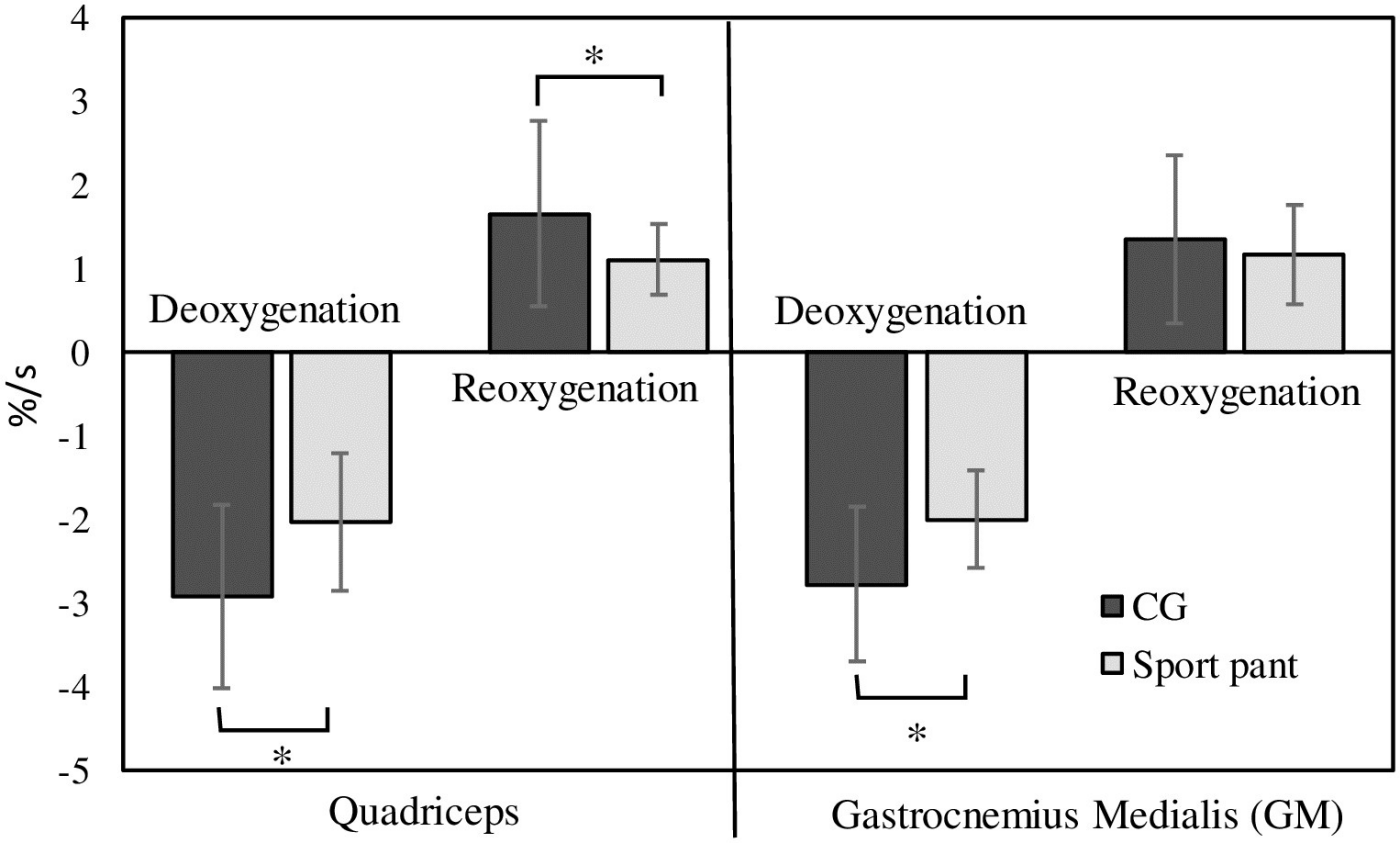
Comparisons of deoxygenation and reoxygenation rates in quadriceps and GM between the garment types. *: Significant difference between CG and sport pants use; CG, compression garment.

Fig 5 presents the comparisons of deoxygenation and reoxygenation rates between garment types. Faster deoxygenation (t = 4.80, *p* = 0.001) and reoxygenation (t = 2.69, *p* = 0.021) rates were achieved with CG use relative to SP use for the quadriceps. For the GM, a faster deoxygenation rate (t = −3.04, *p* = 0.011) was achieved with CG use relative to SP use, but no difference was observed for oxygenation rate (t = 0.85, *p* = 0.412).

Table 2 presents the time course of change in muscle soreness (determined through the VAS). The ANOVA revealed no significant main effect for garment type (quadriceps: F = 2.18, *p* = 0.168; GM: F = 0.70, *p* = 0.794), a significant main effect for time point (quadriceps: F = 32.4, *p* < 0.001; GM: F = 27.5, *p* < 0.001), and no significant interaction (quadriceps: F = 0.441, *p* = 0.818; GM: F = 1.24, *p* = 0.306). VAS values were significantly lower at baseline than at all other time points (*p* < 0.05), and the largest increase was observed at fatigue onset (*p* < 0.05); thereafter VAS values decreased with the increase in rest time (*p* < 0.05).

**Table 2:** Comparisons of muscle soreness (VAS) between garment type and time course of change. (mean±SD).

Time points Parameters	Baseline	Fatigue	Rest 10 min	Rest 20 min	Rest 30 min	Rest 1 hour
Quadriceps						
CG	0.8±0.7#	6.0±2.2	4.3±1.6# ǂ	3.7±1.3# ǂ	3.1±1.4# ǂ	2.4±1.3# ǂ
SP	1.0±1.0	6.4±1.8	4.8±1.8	4.1±1.4	3.4±1.2	2.5±1.4
CG vs SP *p* value	*p* = 0.326	*p* = 0.281	*p* = 0.072	*p* = 0.161	*p* = 0.375	*p* = 0.824
GM						
CG	1.1±1.1#	6.5±2.3	4.5±1.6# ǂ	3.7±1.3# ǂ	3.2±1.4# ǂ	2.6±1.3# ǂ
SP	1.4±1.1	7.1±1.4	4.6±1.7	3.6±1.5	3.1±1.4	2.3±1.6
CG vs SP *p* value	*p* = 0.418	*p* = 0.115	*p* = 0.764	*p* = 0.768	*p* = 0.938	*p* = 0.521

CG, compression garment; SP, sport pants;

*, p<0.05, CG compared with SP;

^#^, p<0.05, compared with fatigue onset;

^ǂ^, p<0.05, compared with baseline; SD, standard deviation.

## Discussion

At baseline, CG use can increase the muscle efficiency for jump height and power by 7% and 4%, respectively, relative to SP use. The vertical jump is an essential skill for many sports, hence vertical jump improvement is the primary athletic training goal [36]. With respect to CG use and jumping, a study reported that compression shorts had no effect on the maximal power of the highest jump, but it enabled participants to maintain power output when performing repeated jumps [37]. Other studies indicated that the power of CMJs is enhanced with the use of compression shorts [38] and lower body CG [39]. A significantly higher single maximal vertical jump (+2.4 cm) was achieved with CG use relative to control conditions [38]; this finding is consistent with that of the present study (+2.3 cm). This could be because the elasticity of the CG increased the propulsive force required for jumping, resulting in a higher jump [38]. This finding indicates that an optimal amount of compression can increase performance, whereas an insufficient or over amount of compression can reduce performance to below the level achieved with loose-fitting pants [40].

CG use reduced the ML displacement of COP by 4.2% relative to the AP displacement and absolute error of JPS. Studies have reported that CG use can enhance the perception of somatosensory information and positively influence balance control by reducing body sway during quiet standing [18, 41, 42]. These results are somewhat inconsistent with those reported in the present study. The beneficial effects of CGs on proprioception control may be most pronounced among older adults, injured individuals [18, 41] and high-level athletes [42]. By comparison, ordinarily active individuals such as healthy and non-injured individuals and non-elite/recreational athletes do not achieve improved proprioception control with CG use [43, 44]. The use of below-knee CGs can improve proprioception of the knee regardless of leg dominance [21]. This favorable effect may be related to the increase in Golgi tendon organ activation and feedback from proprioceptors to muscle [21]. However, lower body CG use may induce negative effects on knee JPS; specifically, it can lead to less JPS relative to non-CG conditions [45].

At baseline, SmO_2_% was higher by 26.4% and 24.6% in the quadriceps and GM, respectively, with CG use relative to SP use. The higher SMO_2_ observed with CG use could be attributed mainly to the changes in skin blood flow. Increasing the external pressure applied to the skin causes an increase in skin blood flow in the area that is subjected to pressure [46]. However, fitting is crucial for CGs because an inappropriate level of compression (excessively tight, high compression or loose, low compression CG) is often associated with negative effects or the absence of effects [8]. The compression tights used in the present study were fitted on the basis of approximate (small, medium, and large) sizes, and an appropriate size was selected for each participant according to his or her anthropometric data. Because variations in anthropometric characteristics and morphology within a population can result in large variations in applied garment pressures [47], current recommendations suggest that CGs must exert a minimum pressure of 17.3 mmHg on the calves and 15.1 mmHg on the thighs [48] to significantly improve venous return. The CGs used in the present study conformed to the aforementioned pressure recommendations (24.4 mmHg on the calves and 16.8 mmHg on the thighs).

The present study revealed that regardless of garment type, fatigue reduced muscle efficacy (jump height and power) and proprioception control, leading to increases in the ML displacements of COP and absolute error of JPS. Moreover, fatigue reduced SmO_2_ and increased muscle soreness (VAS). Nevertheless, delaying fatigue onset should lead to performance maintenance. However, during the performance of exercise-induced muscle fatigue protocol, CG use did not delay fatigue onset. In the present study, the time to fatigue onset with SP and CG use was 51.8 ± 10.2 s and 54.2 ± 9.7 s, respectively; that is, no significant difference between the garment types was observed. Several studies have examined the effect of muscle fatigue on the proprioception of the joints. Some studies have reported that fatigue impairs proprioception [49], whereas others could not identify any effects [50]. Yaggie and Armstrong (2004) revealed that impairments in JPS after fatigue onset could be caused by the reduction of motor neuron output and sensitivity of the muscle afferents groups III and IV [51]. The present study indicates that quadriceps muscle fatigue reduces movement accuracy and increases reconstruction error at the 45° angle of the knee joint. Studies have speculated that CG use enhances JPS by reducing muscle fatigue [21, 22]. However, the results of the present study indicated that CG use failed to reduce the deleterious effects of fatigue on JPS, and absolute error increased immediately after fatigue onset; these findings are consistent with the results of a previous study [52].

Many studies have reported on the relationship between the development of fatigue and the availability and utilization of oxygen during exercise [53], and the suggested that the rate of fatigue development is dependent on the level of muscle oxygenation. However, a study discovered that muscle oxygenation is not always related to a reduction in fatigue levels [54]. Murthy et al. (2001) addressed this controversy by suggesting that the development of fatigue is dependent on muscle oxygenation only when oxygenation levels are reduced by more than 7% [53]. The present study reported a SmO_2_% reduction of up to 68.6% and 58.5% in the quadriceps and GM, respectively after fatigue onset, which indicated that SmO_2_% is highly correlated with the development of fatigue.

Although CG use also failed to reduce the deleterious effects of fatigue on SmO_2_, the SmO_2_ results reflected the dynamic balance between oxygen supply and oxygen consumption in the muscle tissue [16, 55]. The present study revealed that relative to SP use, CG use increased the deoxygenation and reoxygenation rate in the quadriceps by up to 44.3% and 49.5%, respectively. The deoxygenation rate is the negative slope of SmO_2_, which occurred during the fatigue period, a higher deoxygenation rate indicates greater muscle O_2_ demand, and consequently, greater energy consumption [55]. By contrast, a higher reoxygenation rate indicates greater O_2_ delivery relative to O_2_ demand, which increases blood flow during recovery [16]. The rate of phosphocreatine (PCr) resynthesis is related to the reoxygenation rate of SmO_2_ [56], and a higher reoxygenation rate (SmO_2_, rate of recovery) can be advantageous for replenishing PCr stores. As such, a lower body CG may help to sustain anaerobic metabolism during high-intensity exercise by increasing the rate of lactate clearance and delaying the onset of muscular fatigue that accompanies anaerobic activity [4]. By contrast, a longer recovery time is associated with a greater amount of oxygen uptake; hence, slower reoxygenation may be correlated with poor performance [57]. Therefore, an improvement in muscle reoxygenation capacity can improve exercise performance.

Power returned to 92.5% of baseline after 10 min of rest and remained unchanged until the 1-h rest time point, that is, power did not regain its baseline value even after 1 h of rest. Muscle soreness decreased over time after fatigue onset but did not regain its baseline value regardless of CG or SP use even after 1 h of rest. A study explored the effects of CG use on power and muscle soreness at 1, 24, and 48 h after fatigue onset, and it revealed that power and muscle soreness returned to baseline (before exercise) value only after at least 24 h of rest [58]. Another study examined participants who wore a CG for 12 h after performing 100 drop jumps, and it reported that performance recovery for CMJ was significantly improved by CG use at 24 h but not at 1 h after exercise-induced fatigue onset [59]. The ML displacement of COP returned to baseline value by the 10-min rest time point and subsequently decreased until the 1-h rest time point. Relative to SP use, a significantly lower ML displacement was observed with CG use after 20 min of rest. The absolute error of JPS returned to baseline value after 20 min of rest with both CG and SP use, whereas no difference was observed for garment type. Nonetheless, the effect of fatigue was short lived for posture sway, and a study reported that sway velocity decreased to pre-fatigue values after 30 min of rest [60]. Susco et al. (2004) discovered that balance deficits lasted for up to 15 min after fatigue onset, and that balance recovered after the 20 min of rest [61].

SmO_2_ returned to its baseline value after 10 min of rest. The faster reoxygenation rate associated with CG use resulted in the quicker attainment of peak SmO_2_ relative to SP use, after which SmO_2_ was maintained at a high level until the 1-h rest time point; by contrast, with SP use, SmO_2_ increased with time after fatigue onset. A higher SmO_2_ was observed with CG use relative to SP use during the recovery period. Bhambhani et al. (1997) observed rapid increases in muscle oxygenation during the 1–2 min following incremental exercise [62]; this suggests that resting for 1–2 min after entering a recovery period is sufficient and leads to a progressive increase in SmO_2_. Ding et al. (2001) discovered that well-trained athletes exhibit faster recovery for muscle deoxygenation relative to untrained participants [63]. This finding was applied with the use of the lower-body CG in the present study, in which CG use led to a faster recovery relative to SP use. This is a notable finding because the recovery of muscle oxygenation may be an area with crucial implications.

The limitation of the present study is that our results can only be applied to a specific population, that is, healthy young people who exercise regularly but not competitively.

## Conclusions

The present study indicated that relative to SP use, CG use can promote muscle efficacy (jump height and power), proprioception (for the ML displacement of COP but not for the absolute error of JPS), and SmO_2_. A higher deoxygenation rate during the fatigue period and a higher reoxygenation rate during the recovery period were observed with CG use relative to SP use. The higher reoxygenation rate indicates greater O_2_ delivery relative to O_2_ demand, which increases greater blood flow during recovery. SmO_2_ and the ML displacement of COP returned to baseline value after 10 min of rest; by contrast, power and muscle soreness did not return to their baseline values even after 1 h of rest following fatigue onset. The results suggested that SmO_2_ and proprioception can recover after 10 min of rest, however, recovery relating to muscle efficacy and soreness may require at least 24 h or rest regardless of CG or SP use.

## Supporting information

S1 Data (XLS)
